# The Compared Efficiency of the Traditional Method, Radiography without Contrast and Radiography with Contrast in the Determination of Infestation by Weevil (*Sitophilus zeamais*) in Maize Seeds

**DOI:** 10.3390/insects10060156

**Published:** 2019-06-01

**Authors:** Maria Laene Moreira de Carvalho, Eva Rezende Leite, Geraldo Andrade Carvalho, Fabiano França-Silva, Dayliane Bernardes de Andrade, Elizabeth Rosemeire Marques

**Affiliations:** 1 Department of Agriculture, Universidade Federal de Lavras-UFLA, P.O. Box 3037, Lavras 37200-000, Minas Gerais, Brazil; mlaene@gmail.com (M.L.M.d.C.); evarezendeleite@hotmail.com (E.R.L.); dayliandrade@gmail.com (D.B.d.A.); bethagro@yahoo.com.br (E.R.M.); 2 Department of Entomology, Universidade Federal de Lavras-UFLA, P.O. Box 3037, Lavras 37200-000, Minas Gerais, Brazil; gacarval@ufla.br

**Keywords:** levels of infestation, seed damage, sampling, storage, *Zea mays*

## Abstract

Technologies that increase safety and efficiency, while facilitating and streamlining the work of seed analysts, are increasingly required by the seed industry. X-ray image analysis is a technique that has been used in the analysis of grain and seeds because it is fast, accurate and non-destructive. The traditional method to verify the presence of insect damage in seeds involves manual cutting of the seeds, which endangers the safety of the analyst and is time-consuming and repetitive work that leads to visual fatigue. The objective of this study was to compared the efficiency of radiographic analysis with and without contrast in the determination of infestation by *Sitophilus zeamais* Motschulsky (Coleoptera: Curculionidae), at different stages of development, in maize seeds, compared to the traditional method required by seed legislation, which consists of cutting and visual evaluation. Seeds were evaluated regarding the presence of eggs/oviposition signs, larvae, pupae, adult insects, insect damage in five infestation periods (5, 18, 33 and 35 days after infestation), while evaluating the total number of seeds infested, comparing the three methods. For characterization of the oviposition stage, the use of contrast was best at all times of infestation. For the larval stage, there was no difference between the evaluation methods; however, at 18 days, larger infestations were observed by the traditional method. At 5 days, the identification of pupae was better by the traditional method and radiography without contrast, while for the identification of adult insects the best method was the use of radiography without contrast. The characterization of the level of infestation with maize weevil damage was best verified using contrast radiography. Radiographic analysis is efficient in the detection of damage caused by *S. zeamais* in maize seeds. This method of radiographic analysis (with or without contrast) is thus an auxiliary tool to assess the damage and presence of *S. zeamais* in maize seeds.

## 1. Introduction

The growing commercial demand for high quality seeds, whether for internal or external markets, has encouraged producer companies to invest in technologies that ensure minimum seed quality standards in a fast, safe and effective manner [1].

It is important to note that there is great concern about the quality of maize seed produced, since the crop is susceptible to pest insects, especially maize weevil (*Sitophilus zeamais* Mots., 1855; Coleoptera: Curculionidae), which is also responsible for attacking seeds and cereal grains like wheat, rice and sorghum [2,3]. This concern is even more important when considering the ability of maize weevil to disseminate fungi of the genera *Aspergillus, Penicillium and Fusarium* in the grain and seed masses. These fungi take advantage of the insect damage, increasing aflotoxins that contribute to the reduction of germination, vigor and lot disposal in the case of seeds [2,4,5,6].

Rapid and early detection of cereal grain infestation to avoid losses of grain mass and quality in the case of seeds is particularly important, considering that damage to the embryo or reduction of seed stocks can make planting unfeasible.

Studies on insect detection, and grain and seed damage, have been conducted to compare simpler methods, such as manual inspection, sieving, flotation-flotation, and Berlese funnels, which are not always efficient for accurate detection but are necessary when measuring seed marketing standards. More complex research, such as acoustic grain variability with and without damage [7], or chemical analysis, near-infrared spectroscopy [8] and X-ray methods, have the potential for use on an industrial level to detect insects in grain and seed samples. Their usefulness has been demonstrated in research laboratories, according to Neethirajan et al. [9] and Karunakaran et al. [10], who reported more than 95% accuracy using a digital X-ray system, including a classification algorithm, for inspecting wheat for weevils. The technique of radiographic analysis has been highlighted due to its simplicity and reproducibility. It is also non-destructive, can be used to identify full, empty, damaged and broken seeds [11], and also evaluates the damage caused by insects in their different stages of development [12,13].

In Brazil, the usual method for the examination of insect-infested seeds in certified corn lots is performed with the aid of sharp objects and is considered a destructive, time-consuming method, with risks for the health of analysts. A viable alternative is the use of radiographic analysis to detect insect-infested seeds, but it is hypothesized that this technique does not detect all signs of infestation caused by maize weevil, as indeed is the case with the traditional method. It is possible that an adaptation in the methodology of acquiring radiographs of corn seeds from the use of chloroform vapor would allow images of seeds with contrasts to be acquired with high enough quality. Consequently, the efficiency of the analysis would increase, since chloroform vapor penetrates the damaged tissues of the seed and makes them scintillating white in the radiographic images. The use of radiographs with contrast in seeds was mentioned only in the work of Simak, M. [14] for analysis of Pinus seeds (*Pinus sylvestris* L.).

Thus, the objective of this study was to compare efficiency of the traditional method, radiographic analysis without contrast and radiography with contrast, in the determination of infestation by *S. zeamais* at different stages of development, in maize seeds.

## 2. Materials and Methods

To evaluate the efficiency of radiographic analysis in the detection of damage and characterization of the different stages of development of *S. zeamais* in maize seeds, the experiment was conducted in the Laboratory of Seed Analysis of the Department of Agriculture of the Federal University of Lavras.

The insects of *S. zeamais* used in this work were obtained from the Laboratory of Ecotoxicology and Integrated Pest Management (IPM) of the Department of Entomology of the Federal University of Lavras. They were kept in six glass containers, with a capacity of 3000 mL, and in each one were placed 420 seeds of maize and 10 pairs of adult insects. The containers were closed with voile tissue to ensure air circulation and the survival of insects. The containers were kept in a climate chamber at 25 ± 2 °C, 65% relative humidity (RH) without photophase, for a period of two months.

At 5, 18, 33 and 35 days after infestation, characterization evaluations of insect development stages and a comparison between traditional methods and radiographic analysis were performed for the purpose of examining infested seeds. The respective periods of infestation were defined according to the stages of development of *S. zeamais*, in which the period of five days corresponds to the average time of embryonic development; 18 days is the mean period of the larval phase; 33 days is the median time of the pupal stage; and 35 days is the average duration of the biological cycle (from egg to adult insect).

### 2.1. X-Ray Test: Radiography without Contrast

To obtain radiography without contrast, in each evaluation period (5, 18, 33 and 35 days), 100 seeds were removed from the glass pot, numbered one by one and distributed equally, with the side closest to the embryo turned upwards, in a single layer, on eight transparent acrylic slides with the capacity for 25 seeds each. The seeds were numbered according to the position on the plate to enable their identification. The material was then subjected to X-ray testing at 24 KV intensity for 60 s at 35 cm from the emitting source, using X-ray apparatus (MX-20 Faxitron, Lincolnshire, IL, USA) to obtain radiographs. The images obtained were analyzed visually and the percentage of seeds with the presence of the insect at any stage of development were counted.

### 2.2. X-Ray Test: Radiography with Contrast

In order to obtain radiography with contrast, the seeds that were on the plates used in the previous step were placed in hermetically sealed glass containers, containing a metal handle with cotton wool soaked in chloroform solution (CHCl_3_), to penetrate the damaged parts of the seeds and thus generate contrast in the X-rays [14]. After 2 h of indirect seed/chloroform exposure, the transparency plates were removed and subjected to X-ray testing using the settings described in the previous step. The images obtained were analyzed visually, and the percentage of seeds with the presence of the insect at any stage of development were counted.

### 2.3. Analysis of Infested Seeds by the Traditional Method

After contrast radiography, the seeds were removed from the transparency plates and distributed one by one in cryogenic tube boxes, with a capacity of 100 compartments. Each compartment containing the seed was filled with water for 24 h to soften the tegument and favor the cutting of the seeds. After the immersion time and with the aid of a scalpel, the seeds were cut to verify the presence of eggs, larvae, pupae and adults of the insect [15]. The results of the observed infestation were expressed as a percentage.

Once cut, the seeds were placed on Ethylene Vinyl Acetate (EVA) blue rubber used as a background for the pictures and photographed with a Sony α 57 DT 18–55 mm F3.5–5.6 SAM camera. Subsequently, the images of the photographed seeds were compared with those of the X-rayed seeds, with and without contrast.

### 2.4. The Design Used in Tests and Statistical Analysis

A completely randomized design with 6 replicates of 100 seeds was used, with a subdivided plot; the infestation period was in the plot (5, 18, 33 and 35 days of infestation), and the method of analysis was in the subplot (traditional, X-rays without contrast and X-rays with contrast), totaling 12 treatments. Data of the observed infestation of weevil in maize seeds were analyzed by a one-way analysis of variance (ANOVA). Where significant differences were revealed (F-ratio, *p* < 0.05), a stepwise general linear models (GLM) procedure was used for the Tukey-Kramer multiple comparisons test to determine the pattern of differences between methods. Significant differences between methods for determination of infestation by weevil in maize seeds were concluded when the coefficient of the interaction term was significant at *p* < 0.05. Additionally, standard deviations (SD’s) were calculated and used as means separation tests. Analyses were performed with the SAS^®^ program (version 9.4, SAS Institute, Cary, NC, USA).

## 3. Results

**Figure 1:**
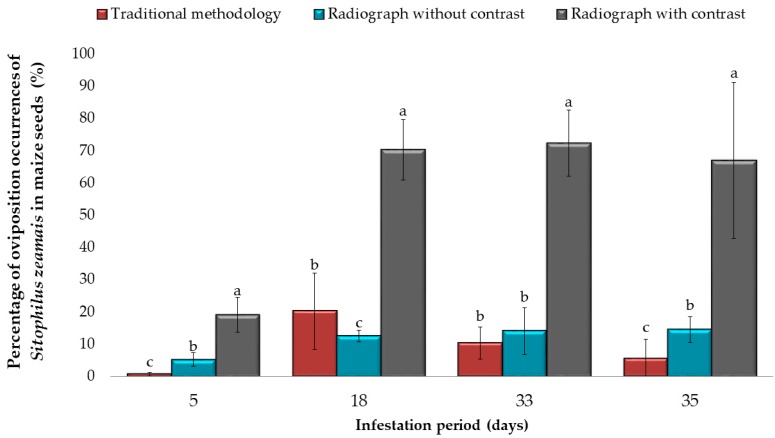
Detected oviposition occurrences of *Sitophilus zeamais* in maize seeds at 5, 18, 33 and 35 days after infestation by traditional method, radiograph without contrast and radiograph with contrast. The bar graph shows the means and the error bars show the ± standard deviations (SD) in each period of infestation (Tukey-Kramer Test, *p* < 0.05).

The detection of the percentage of oviposition of *S. zeamais* in maize seeds varied according to the evaluation period and the method of analysis of the infestation (Figure 1). Within the infestation times (5, 18, 33 and 35 days after infestation), the radiographic method of contrast analysis of the infested seeds differed significantly (*p* < 0.001) from the traditional methods (cutting the seeds with scalpel) and the radiography without contrast. The sensitivity of the radiographic analysis method with contrast allowed inference of the approximate occurrence of 20 to 70% of corn seeds with corn weevil oviposition signs.

**Figure 2:**
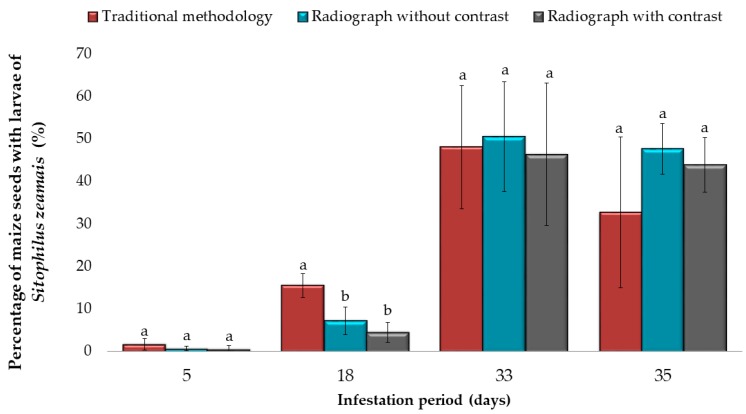
Average percentage of maize seeds infested with larvae of *Sitophilus zeamais* at 5, 18, 33 and 35 days after infestation, by traditional methods, radiograph without contrast and radiograph with contrast. The bar graph shows the means, and the error bars show the ± SD in each period of infestation (Tukey-Kramer Test, *p* < 0.05).

In the analysis of maize seeds infested by the weevil, larvae were detected internally in the seeds. Significant differences (*p* < 0.001) were found between the different methods for infested seed analysis, with the traditional method being the most sensitive in detecting almost 20% of seeds infested by larvae (Figure 2). For the other infestation periods (5, 33 and 35 days after exposure), there were no significant differences (*p* > 0.05) between the three methods evaluated, which detected 5% to 50% of infestation larvae of *S. zeamais*.

In relation to the determination of the presence of maize seeds infested by pupae and adult insects of maize weevil, infestations were observed only at 5 days of evaluation, with significant differences between the three evaluation methods (*p* < 0.001) (Table 1). For the pupal stage, the traditional method was more sensitive when compared to radiography with contrast. For the adult insect stage of *S. zeamais*, the radiography without contrast showed a higher number of infested corn seeds, followed by radiography with contrast and the traditional method, which involves cutting the seeds.

**Figure 3:**
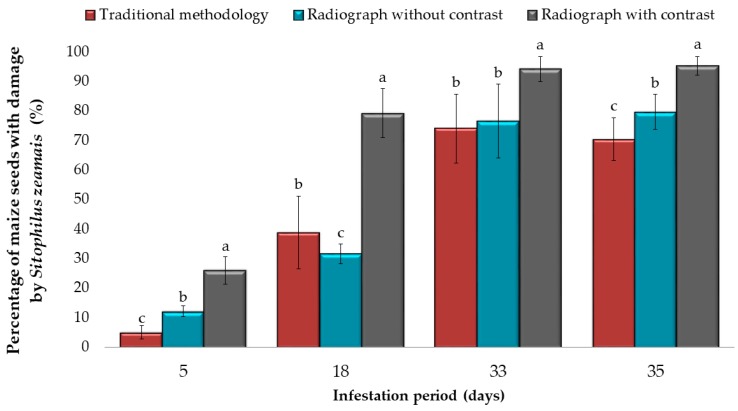
Average percentage of maize seeds with damage by *Sitophilus zeamais* in different assessment periods, assessed by traditional methods, radiograph without contrast and radiograph with contrast. The bar graph shows the means, and the error bars show the ± SD in each period of infestation (Tukey-Kramer Test, *p* < 0.05).

In relation to the presence of *S. zeamais* damage in maize seeds, significant differences (*p* < 0.001) were observed in the total number of seeds damaged by the weevil, comparing the three methods of analysis (Figure 3). Although traditional methods and radiography without contrast varied according to the infestation period (5, 18, 33 and 35 days after exposure), the radiography with contrast method showed greater sensitivity in detecting damage (up to 90% of seeds damaged by weevil), often imperceptible by traditional methods and radiography without contrast.

**Figure 4:**
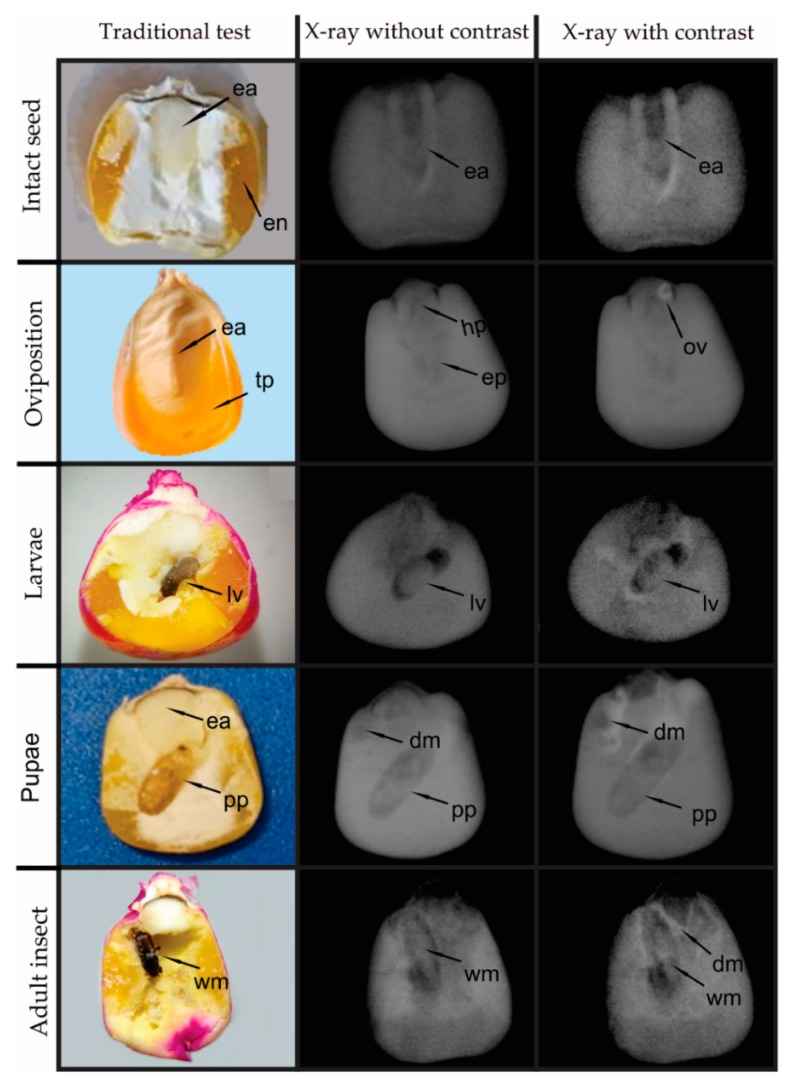
Characterization of the damage (dm) and stages of development of *Sitophilus zeamais how* sign of oviposition (ov), larvae (lv), pupae (pp) and adult insect (wm) inside corn seeds and seeds with intact structures (embryonic axis (ee), hypocotyl (hp), epicotyl (ep), endocarp (en) and tegument + pericarp (tp)).

After analysis of corn seeds and observation of photo and radiographs without and with contrast (Figure 4), it was verified that in the traditional method with seed cutting and radiography without contrast, it is only possible to identify the embryonic axis (ee), hypocotyl (hp), epicotyl (ep), endocarp (en) and tegument + pericarp (tp), without damage caused by corn weevil. In contrast radiographs, in addition to the structures of the seed, it is possible to detect oviposition (ov) signs that appear on the radiographs as darker points, surrounded by a white scintillating halo. The larvae (lv) are round, with uniform bodies, and on the radiographs with and without contrast they are smaller in relation to their galleries built inside the seeds. In contrast radiographs, the chloroform vapor (chl) marks, with a scintillating white halo, show the seed tissues damaged by the insect. The pupae (pp) are more irregular and oval, of brown color, observed after the cutting of the seeds in the traditional methodology and, in the radiographs, they are verified occupying the whole gallery, with nearby regions presenting a dark gray shade, possibly other damage (dm) caused by the weevil. Finally, the adults of the corn weevil (wm) are smaller in relation to the pupae, and they are dark brown (observation in the traditional methodology). In the X-rays with and without contrast the adult weevils are observed occupying a third of the gallery and positioned at the end near to the exit from the interior of the seeds.

## 4. Discussion

The positive effects of contrast radiography on the evaluation of *S. zeamais* damage and oviposition signs arise from the ability of contrast agent, such as chloroform, to penetrate the damaged areas of the seed, causing differences in tissue density. This makes the areas affected by the insect brighter (light gray), while intact areas of the seed are observed in darker grayscale on the radiograph analyzed. [14,16].

On radiographs without contrast, oviposition signs were circular, dark, tiny, and almost imperceptible, and when observed with contrast, they were surrounded by a white halo (higher density). In earlier research, there are reports that oviposition signs and first instar larvae of *S. zeamais* and *Sitophilus oryzae* (Linnaeus) are often difficult to identify in radiographs [17]. Likewise, difficulties in the identification of eggs of *Oryzaephilus surinamensis* L. (Coleoptera: Silvanidae) on date seeds analyzed by radiographs have also been reported [18]. In the aforementioned works the problem reported was solved by an algorithm developed in MATLAB for segmentation of the radiographs, resulting in the efficient identification of infested and non-infested seeds.

When analyzing wheat radiographs after using Adobe Photoshop and MicroImage to obtain contrast, Fornal et al. [12] accurately detected eggs of *Sitophilus granarius* (L.) in the grains five days after oviposition. Chelladurai et al. [19] combined X-ray features with near-infrared (NIR) hyperspectral characteristics and, in comparison to isolated X-rays, reached greater accuracy in the identification of *Callosobruchus maculatus* (Fabr.) (Coleoptera: Bruchidae) eggs and larvae in soybean seeds. However, they pointed out the possible non-viability of these systems, given the increase in cost and time for the detection of infestation.

Regarding the use of X-rays without contrast, there are reports of the efficiency of the method in *S. granarius* characterization and its damage to wheat grains [20]; correct classification of damage by bedbugs in cotyledons of *Crotalaria juncea* L. (Fabaceae) [21]; distribution mapping, interaction and competitive behavior among the larvae of *S. zeamais* in maize seed [22]; detection of popcorn maize seed infestation by *S. zeamais* and classification into categories: small larvae, medium-sized larvae, pupae and adults [23], and the finding of the infestation of *Cryptocarya aschersoniana* Mez. (Lauraceae) by insects of the family Curculionidae, through radiographic analysis [24].

A probable explanation for the results obtained in the aforementioned works is that the X-rays form different levels of attenuation of the objects (seed/insect) X-rayed. This attenuation is obtained by reducing the intensity of X-rays as they pass through objects of various densities [25]. Thus, a less dense medium, such as damage and oviposition signs, is displayed in black on the radiographs, while a denser medium, such as undamaged seeds, is highlighted in white. However, due to the natural existence of cavities in the seeds that cause darker areas, errors are likely to occur in the classification of seeds, whether infested or not [18].

As regards the use of contrasting solutions, such as chloroform, these increase the differentiation between tissues and allow the evaluation of the structures and integrity of the tissues, but they are costly and possibly carcinogenic due to high concentrations and doses of the product. These characteristics, associated with the existence of plant species that react differently to the same substance [26], result in limiting factors for chloroform solution use in routine laboratories.

## 5. Conclusions

The use of contrast radiography showed greater sensitivity in the detection of damage and percentage of infestation of *S. zeamais* at different stages of development in corn seeds. The method of radiographic analysis without use of contrast (chloroform), followed by the traditional method, with seeds cut using a scalpel, were less sensitive to the detection of weevil infestation in corn seeds, underestimating the presence of oviposition signs and other damage. In addition to this disadvantage, the slowness of the traditional method leads to the conclusion that both radiography without contrast and radiography with contrast are appropriate tools for the examination of insect-infested seeds. Future studies are needed to study mechanisms to improve the efficiency of radiographic analysis in identifying other insect pests infesting different seed species.

## Figures and Tables

**Table 1:** Comparison of the traditional method with scalpel (**T**) without contrast radiography (**S**) and with contrast radiography (**C**) in relation to the evaluation of the presence of adult insects of *Sitophilus zeamais* causing damage in maize seeds after 5 days of infestation.

Stages of Development	5 Days after Infestation					
	T		S		C	
	Mean	Standard Deviation	Mean	Standard Deviation	Mean	Standard Deviation
% Pupae	2.21 a	2.47	1.67 ab	2.06	1.29 b	1.71
% Adult insects	8.29 c	4.62	16.21 a	9.05	12.42 b	4.97

Values within the same line followed by the same letter are not significantly different, Tukey-Kramer (*p* < 0.05).
